# The Curious Anti-Pathology of the *Wld*^*s*^ Mutation: Paradoxical Postsynaptic Spine Growth Accompanies Delayed Presynaptic Wallerian Degeneration

**DOI:** 10.3389/fnmol.2021.735919

**Published:** 2021-09-10

**Authors:** Oswald Steward, Jennifer M. Yonan, Paula M. Falk

**Affiliations:** ^1^Reeve-Irvine Research Center, University of California, Irvine, Irvine, CA, United States; ^2^Department of Anatomy & Neurobiology, University of California, Irvine, Irvine, CA, United States; ^3^Department of Neurobiology and Behavior, University of California, Irvine, Irvine, CA, United States; ^4^Department of Neurosurgery, University of California, Irvine, Irvine, CA, United States; ^5^Department of Neuroscience, University of Virginia, Charlottesville, VA, United States

**Keywords:** nicotinamide adenyltransferase 1 (NMNAT1), ubiquitination factor U4B, denervation, reinnervation, dentate gyrus, entorhinal cortex, perforant path

## Abstract

The *Wld*^*s*^ mutation, which arose spontaneously in C57Bl/6 mice, remarkably delays the onset of Wallerian degeneration of axons. This remarkable phenotype has transformed our understanding of mechanisms contributing to survival vs. degeneration of mammalian axons after separation from their cell bodies. Although there are numerous studies of how the *Wld*^*s*^ mutation affects axon degeneration, especially in the peripheral nervous system, less is known about how the mutation affects degeneration of CNS synapses. Here, using electron microscopy, we explore how the *Wld*^*s*^ mutation affects synaptic terminal degeneration and withering and re-growth of dendritic spines on dentate granule cells following lesions of perforant path inputs from the entorhinal cortex. Our results reveal that substantial delays in the timing of synapse degeneration in *Wld*^*s*^ mice are accompanied by paradoxical hypertrophy of spine heads with enlargement of post-synaptic membrane specializations (PSDs) and development of spinules. These increases in the complexity of spine morphology are similar to what is seen following induction of long-term potentiation (LTP). Robust and paradoxical spine growth suggests yet to be characterized signaling processes between amputated but non-degenerating axons and their postsynaptic targets.

## Introduction

The remarkable phenotype of delayed Wallerian degeneration in mice carrying the *Wld*^*s*^ mutation has dramatically revised our understanding of mechanisms contributing to survival vs. degeneration of mammalian axons after separation from their cell bodies. The *Wld*^*s*^ mutation occurred spontaneously in a colony of C57Bl/6J mice at the Olac breeding colony in Bicester England, and was discovered because of its signature effect—axons that are separated from their cell bodies appear intact at the light and electron microscopic level for many days, and are able to conduct action potentials for up to 2 weeks (Lunn et al., 1989; Perry et al., 1990, 1991). Herein, we use the term “orphaned axons” to refer to axons that have been separated from their parental cell body that survive. The prolonged viability of orphaned axons in *Wld*^*s*^ mutant mice is in dramatic contrast to the situation in normal mice, where amputated axons exhibit signs of degeneration within hours. The dramatic survival of orphaned axons in *Wld*^*s*^ mice motivated studies to identify and target the relevant molecular pathways to abrogate axonal degeneration in various neurodegenerative diseases (Fischer et al., 2005; Gillingwater et al., 2006; Coleman and Freeman, 2010).

Early genetic studies revealed that the mutant *Wld*^*s*^ gene is dominant (Perry et al., 1990), indicating a gain of function. Subsequent studies revealed that the mutant locus encodes a fusion protein made up of the amino terminal 70 amino acids (N70) fragment of ubiquitination factor U4B, the complete coding region of NAD+ synthesizing enzyme nicotinamide adenyltransferase 1 (NMNAT1), and an 18 amino acid linking region (Mack et al., 2001). Enzymatically active NMNAT1 is critical for the axon survival phenotype (Avery et al., 2009). Although the mutant protein accumulates in the nucleus, studies involving fusion of the *Wld*^*s*^ protein with axonally transported proteins reveal that axonal targeting is both necessary and sufficient to confer protection against Wallerian degeneration (Babetto et al., 2010). Insights into mechanisms came from subsequent studies revealing that NMNAT2, a rapidly turning over protein related to NMNAT1, is a natural survival factor for axons and that axonal delivery of the *Wld*^*s*^ protein with enzymatically active NMNAT1 can substitute for NMNAT2 to maintain axonal NAD levels after injury and promote axonal survival [for a recent review, see (Coleman and Hoke, 2020)].

A deeper understanding of the mechanisms underlying axonal degeneration came as a result of the discovery of the pro-axon degeneration molecule SARM1 [for a review, see (Figley and DiAntonio, 2020)]. Initial studies revealed that loss of the protein SARM1 in flies or mice conferred protection against axon degeneration similar to that of the *Wld*^*s*^ mutation. Subsequent studies revealed that SARM1 degrades NAD and that this NADase activity is essential for SARM1 to promote degeneration. A relationship between SARM1 and NMNAT was revealed by the finding that in healthy axons, NMNAT2 protects axons from SARM1-mediated degeneration, and loss of NMNAT2 as a result of injury activates SARM1, triggering degeneration. Moreover, over-expression of and axonal targeting of NMNAT1 blocks injury-induced SARM1 activation and NAD degradation critical for axon degeneration (Sasaki et al., 2016). Together, these studies revealed that axon degeneration is an active process regulated by the balance of action of pro- and anti-degenerative molecular pathways. These findings reveal potential targets for therapeutic interventions to prevent or delay axon degeneration in various neurodegenerative diseases (Coleman and Hoke, 2020; Figley and DiAntonio, 2020).

Although numerous studies reveal how the *Wld*^*s*^ mutation affects axon degeneration, especially in the peripheral nervous system, less is known about how the mutation affects degeneration of synapses in the mammalian CNS. In this regard, electron microscopic studies can provide important clues about cell biological mechanisms. One electron microscopic study (Gillingwater et al., 2006) reported that degeneration of cortico-striate synapses after cortical lesions was dramatically delayed in *Wld*^*s*^ mice, but orphaned synapses began to exhibit ultrastructural changes similar to degenerating synapses in control mice by 8–10 days post-lesion. Gillingwater et al. (2006) did not assess the fate of synaptic terminals in *Wld*^*s*^ mice at time points after 10 days post-lesion.

Although no unique features of orphaned presynaptic terminals in *Wld*^*s*^ were detected by Gillingwater et al. (2006), a notable finding was an increased incidence of large, complex spines with multiple segmented postsynaptic densities (PSDs). It was suggested that these complex synaptic profiles reflected an enhanced plastic response at both injured and uninjured synapses, but exactly what this involves was not defined. Also, because this study did not assess time points beyond 10 days post-lesion, it was unknown whether spine alterations were transient or permanent.

To further explore how the how the *Wld*^*s*^ mutation affects CNS synapse degeneration, spine morphology and synaptic plasticity, here we use electron microscopy to characterize synaptic degeneration and replacement on dentate granule cells following lesions of perforant path inputs from the entorhinal cortex. This model of post-lesion synaptic plasticity has been extensively studied in rats (Matthews et al., 1976a,b; Steward and Vinsant, 1983) and mice (Perederiy et al., 2013). In wild type rats and mice, loss and reformation of synapses is accompanied by withering and re-growth of dendrites and loss and re-appearance of dendritic spines (Caceres and Steward, 1983; Steward et al., 1988a). Electron microscopic studies indicate that loss of spines actually involves a collapse or withdrawal of the spine into the parent dendrite (Steward and Vinsant, 1983). This model of lesion-induced synapse turnover provides a unique opportunity to explore whether there are unique ultrastructural features of synaptic terminals on orphaned CNS axons and how the mutation affects structural adjustments of denervated postsynaptic neurons. Of particular interest is whether loss and regrowth of dendritic spines spine occurs with the same delay as the onset of terminal degeneration in *Wld*^*s*^ mice, or whether the mutation also confers a degree of protection on the post-synaptic side, preventing or delaying spine atrophy.

Our quantitative electron microscopic analysis addresses two questions: (1) What is the timing of degeneration of orphaned synaptic terminals in *Wld*^*s*^ mice? (2) What changes occur in spines contacted by orphaned synapses, but are not exhibiting signs of degeneration?

Our results provide a thorough characterization of the timing of synapse degeneration in *Wld*^*s*^ mice and identify previously uncharacterized early ultrastructural changes prior to the time that orphaned synapses begin to exhibit typical signs of Wallerian degeneration. In addition, we document striking alterations in the structure of dendritic spines contacted by synapses of orphaned axons including dramatic hypertrophy of spine heads, enlargement and segmentation post-synaptic membrane specializations (PSDs), and development of spinules. These increases in the complexity of spine morphology are very similar to what is seen following intense synaptic activity and induction of LTP [for a recent review, see (Petralia et al., 2021)]. Robust and paradoxical spine growth during the time period when spines in WT mice are withering and retracting suggests yet to be characterized signaling processes between orphaned axons and their postsynaptic targets.

## Materials and Methods

### Experimental Animals and Surgical Procedures

C57Bl/6 mice were purchased from Jackson Labs. *Wld*^*s*^ mice were obtained from our local breeding colony, originally established from *Wld*^*s*^ mice on a C57Bl/6 background obtained from Jackson labs. All procedures were approved by the Institutional Animal Care and Use Committee (IACUC) of the University of Virginia.

### Entorhinal Cortex Lesions

For surgery, adult male mice (3–6 months of age) were anesthetized with Avertin (2.5 μg tribromoethanol per 10 g of body weight). The fur was removed from the area overlying the posterior cortex, and the scalp was incised. The skull overlying the entorhinal area was removed and the posterior portion of the cerebral hemisphere was removed by gentle aspiration. The scalp was sutured and mice were allowed to survive for 4, 8, 12, and 16 days post-lesion. The extent of the lesion was assessed by histological analysis. Damage sometimes extended slightly into the mesencephalon and diencephalon, but when lesions extended into the hippocampus or dentate gurus, the animals were excluded. Quantitative analyses were carried out on mice with acceptable lesions (at least 3 mice per genotype per timepoint).

### Tissue Preparation

Mice were perfused transcardially with 2% paraformaldehyde/2% glutaraldehyde in 0.13 M cacodylate buffer, pH 7.2. Brains were blocked by a coronal cut approximately 2.5 mm posterior to bregma. Coronal sections were cut by hand from the rostral block containing the dorsal hippocampus, and sample blocks were dissected from the dorsal blade of the dentate gyrus approximately 1.5–2.0 mm posterior to bregma. The posterior portion of the brain containing the entorhinal cortex was embedded in egg yolk and sectioned in the horizontal plane; sections were stained for Cresyl violet for lesion assessment.

Blocks containing the dentate gyrus were osmicated and embedded in plastic. Thin sections were cut using an LKB ultra-microtome perpendicular to the layer of the granule cell bodies in order to obtain sections that extend from the cell body layer to the zone containing the tips of the dendrites (the outer molecular layer of the dentate gyrus). Sections were taken at 1 μm and stained with toluidine blue to aid with mapping in the EM; sections cut at approximately 600 Å were mounted on grids and stained with uranyl acetate/lead citrate.

### Quantitative Electron Microscopic Analyses

We evaluated synapse degeneration and replacement using quantitative electron microscopic techniques as in our previous studies in rats (Steward and Vinsant, 1983). Sample images were taken at 8,000× magnification in the middle molecular layer of the dentate gyrus so as to sample a region encompassing approximately 2,000 μm^2^ of neuropil. From these collections of images, we counted intact synapses and degenerating synapses. Intact synapses were defined as appositions between a presynaptic process with at least three synaptic vesicles and a postsynaptic element with a definable postsynaptic density (PSD). Degenerating synapses were identified as presynaptic terminals with apposed PSD exhibiting either electron dense or electron lucent forms of degeneration. Data are expressed as intact synapses and degenerating synapses/100 μm^2^ of neuropil.

Here, we express data as synapses/100 μm^2^ of neuropil rather than using more sophisticated stereological techniques (such as the physical bisector technique) because this allows data to be compared across labs and with data from previous studies in rats and mice (Matthews et al., 1976a,b; Steward and Vinsant, 1983). Although stereological techniques are preferred when determining absolute synapse numbers, they are not essential for measurements of changes in relative numbers of synapses over time (for example, as in the present study) providing that the size of the elements being counted is relatively constant over time. In this regard, our previous study in rats revealed no changes in the size of presynaptic terminals or synapses over time post-injury (Steward and Vinsant, 1983).

### Image Manipulation

One electron micrograph was manipulated to cover a scratch (Figure 4C). Some EM images are colorized to highlight key structures.

**FIGURE 1:**
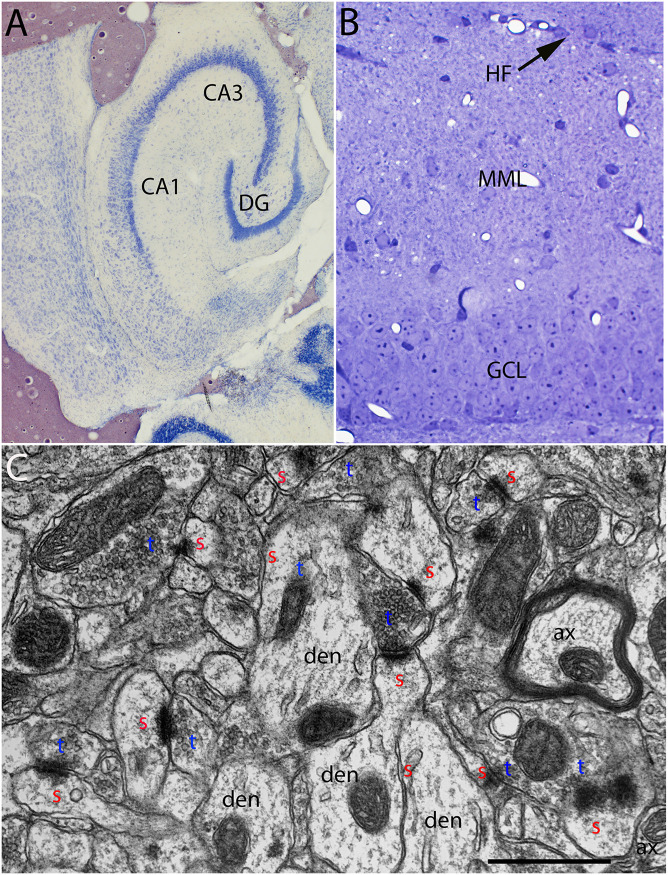
**(A)** Cresyl violet stained horizontal section illustrating an example of the entorhinal cortex lesions. **(B)** Toluidine blue-stained section illustrating the dorsal blade of the dentate gyrus and the area containing degenerating synapses in the outer molecular layer. **(C)** Normal ultrastructure of synapses in the middle molecular layer of the dentate gyrus. CA1–CA3, subdivisions of the hippocampus; DG, dentate gyrus; HF, hippocampal fissure, the boundary between the distal dendrites of granule cells and distal dendrites of hippocampal neurons in CA1; MML, middle molecular layer; GCL, granule cell layer; red s, spine head; blue t, synaptic terminal; den, dendrite; ax, myelinated axon.

**FIGURE 2:**
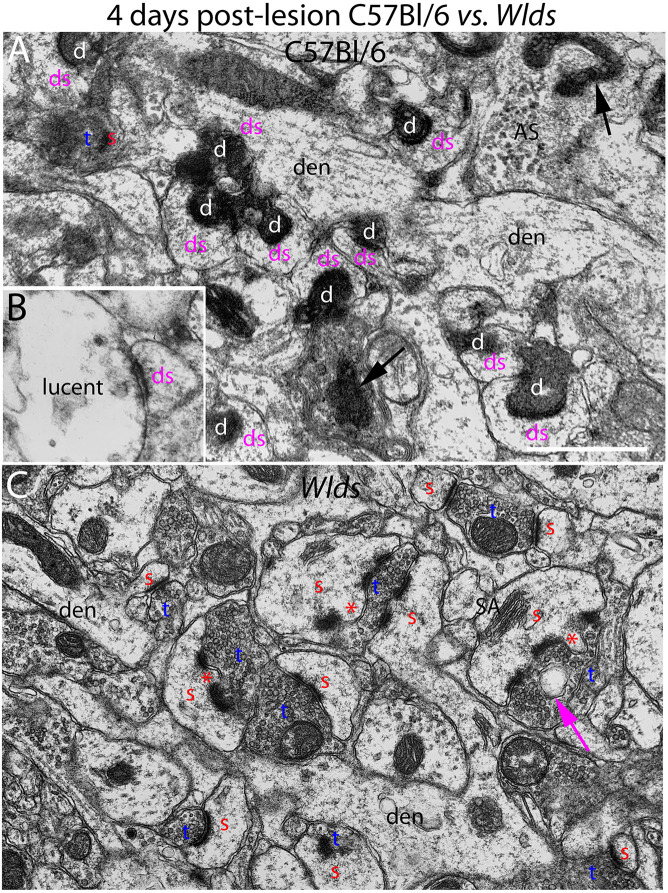
Ultrastructure of synapses at 4 days post-lesion in C57Bl/6 and *Wld*^*s*^ mice. **(A)** Synapses in the middle molecular layer exhibiting typical dark-dense degeneration at 4 days post-lesion in C57Bl/6 mice. Spines contacted by degenerating presynaptic terminals had abnormal morphologies. **(B)** Presynaptic terminal exhibiting the lucent form of degeneration. **(C)** Non-degenerating presynaptic terminals in the middle molecular of *Wld*^*s*^ mice at 4 days postlesion. Spine heads are large and some have multiple PSDs and some had finger-like protrusions (termed spinules) that extended into the presynaptic terminal (marked by red asterisks). Large complex spine apparatuses (SA) were also evident. Red s, spine head contacted by non-degenerating synapse; Blue t, non-degenerating synaptic terminal; white d, degenerating synaptic terminal; Purple ds, spine contacted by degenerating presynaptic terminal; den, dendrite; ax, myelinated axon. Calibration bar in **(A)** = 0.25 μm and applies to **(A,B)**.

**FIGURE 3:**
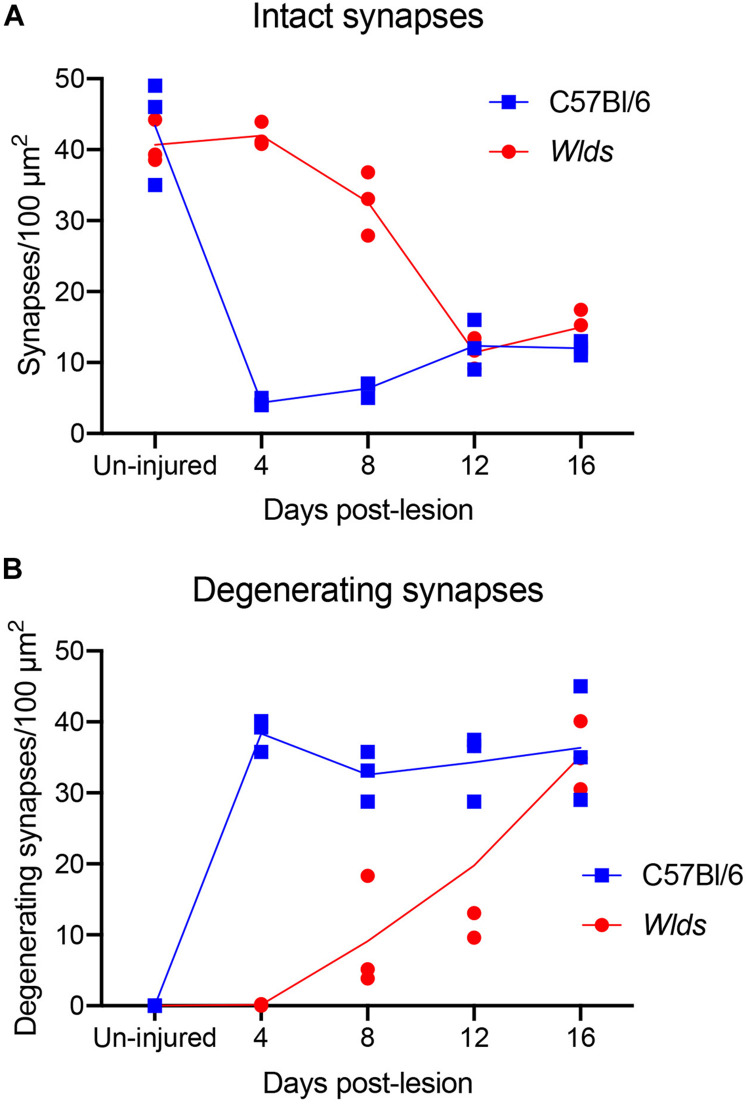
Counts of intact synapses **(A)** and degenerating synapses **(B)** per 100 μm^2^ of neuropil in un-injured C57Bl/6 vs. *Wld*^*s*^ mice at different times post-injury.

**FIGURE 4:**
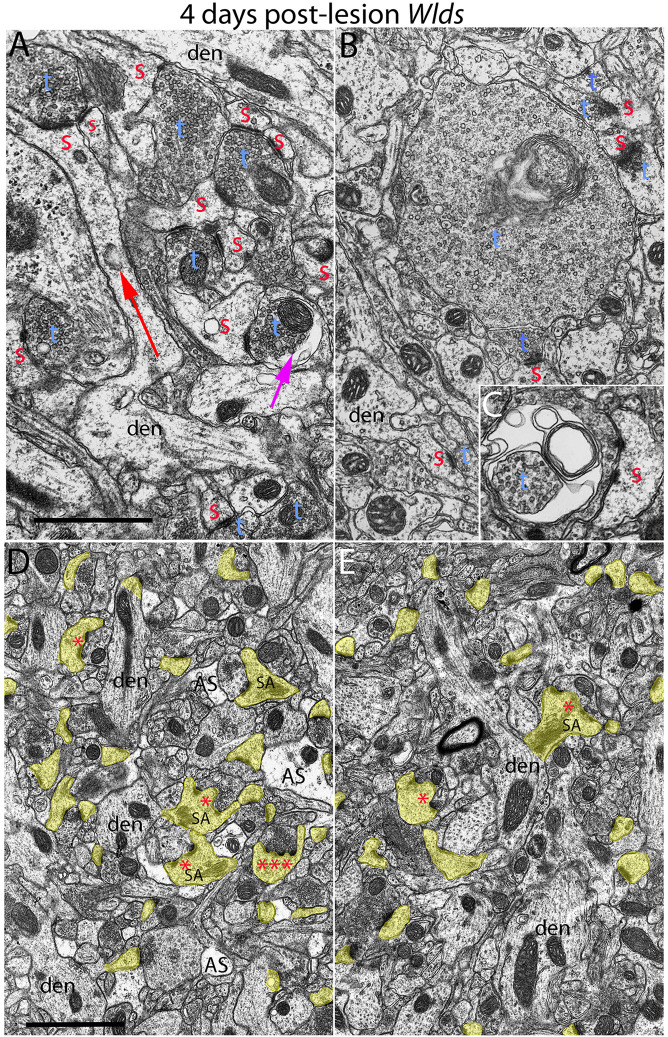
Examples of synapses with abnormal morphologies at 4 days post-lesion in *Wld*^*s*^ mice. **(A)** Synaptic terminal with washed out area (purple arrow) with other normal-appearing synaptic terminals. Note also very long spine neck (red arrow). **(B)** Exceptionally large presynaptic terminal filled with vesicles and complex whorls of membranes. **(C)** Presynaptic terminal with whorls of membranes and a membrane-bound sac containing vesicles. **(D)** Lower magnification view of chaliciform spines in the area of orphaned axons in *Wld*^*s*^ mice (middle molecular layer). Note lighter astrocyte processes (AS) intermingled with synaptic terminals and dendrites. **(E)** Lower magnification view of chaliciform spines in the non-denervated inner molecular layer. Red s, spine head contacted by non-degenerating synapse; Blue t, non-degenerating synaptic terminal; den, dendrite; calibration bar in **(B)** = 0.25 μm and applies to **(A–C)**. Calibration bar in **(D)** = 1 μm and applies to **(D,E)**. *spinule; ***multiple spinules.

## Results

Aspiration lesions of the entorhinal cortex are somewhat variable because they are done without stereotaxic guidance. Accordingly, for the electron microscopic (EM) analysis, we selected cases in which the lesion destroyed most of the entorhinal cortex (both medial and lateral divisions) with little or no damage to the posterior hippocampus. Figure 1A illustrates an example of the lesion. Cases where lesions were incomplete or where there was damage to the posterior hippocampus were excluded.

From the collection of cases with acceptable lesions, at least 3 mice of each strain were prepared for EM at each timepoint post-lesion. One μm sections from the block were stained with toluidine blue to aid in mapping in the electron microscope (Figure 1B). The area of the molecular layer containing degenerating axons and synapses was easily identifiable in the toluidine blue-stained sections (Figure 1B). In the electron microscope, the section was mapped by recording stage coordinates, the layer of granule cells was identified and the location of the hippocampal fissure was identified. Non-overlapping sample images were taken at 8,000× in the middle portion of the molecular layer of the dorsal blade of the dentate gyrus avoiding large blood vessels and flaws in the section (knife marks for example).

### Normal Ultrastructure in Un-Operated Mice

The ultrastructural appearance of synapses in the molecular layer of the dentate gyrus in un-operated mice was not noticeably different from what has been previously described in rats (see Figure 1C). Also, the ultrastructural appearance of synapses in *Wld*^*s*^ was unremarkable and was not noticeably different than control C57Bl/6 mice. Counts of synapses in uninjured mice revealed that synapse density was comparable in the two strains (43.3 ± 6/100 μm^2^ in C57Bl/6 mice, 40.6±2.4/100μm^2^ in *Wld*^*s*^ mice).

### 4 Days Post-lesion

At 4 days post-lesion, most synapses in the denervated zone exhibited clear signs of degeneration in C57Bl/6 mice. Most presynaptic terminals exhibited the typical dark-dense form of degeneration in which the contents of the terminal appeared electron dense and compressed; synaptic vesicles are not seen in these types of degenerating terminals (Figure 2A). A small number of synapses (average of 1.5% of the total number of degenerating synapses) exhibited the lucent form of degeneration in which the contents of the presynaptic terminal appear washed out with synaptic vesicles floating in empty space (Figure 2B). The lucent form of terminal degeneration is common at 2 days post-lesion but is less common by 4 days (Steward and Vinsant, 1983). Scattered amongst the degenerating synapses were a few normal-appearing synapses (for quantification, see below). Quantitative analyses revealed that the number of intact synapses per 100 μm^2^ decreased to about 10% of un-operated controls, and the number of degenerating synapses corresponded closely to the number of intact synapses that were lost (Figure 3).

Most spines contacted by degenerating presynaptic terminals had abnormal morphologies. Spine heads were cup-shaped, and spine necks were more amorphous in form in comparison to normal spines. Some degenerating synapses were on irregularly shaped mounds along the dendritic shaft that contained flocculent material similar in appearance to what is seen in spine heads (Figure 2A). The shape and general appearance of these mounds strongly suggests that these were spines that had collapsed or withdrawn into the dendrite. In contrast to dendrites in un-operated mice, which have a generally regular diameter, dendrites in the area of degeneration had an irregular, varicose, lumpy appearance.

A few empty PSD’s were seen at 4 days post-lesion (PSDs that were not apposed by either an intact or degenerating presynaptic terminal). Some of these may be synapses that are sectioned off-center so that the presynaptic element is out of the plane of section, but others represent synapses from which the presynaptic terminal has been removed. Some empty PSDs were apposed by a glial process or slip of membrane, which we interpret as the remnant of the degenerated presynaptic terminal.

In striking contrast, in *Wld*^*s*^ mice at 4 days postlesion, there were virtually no profiles exhibiting the dark-dense form of degeneration (Figures 2C, 4); indeed, only one presynaptic terminal exhibiting electron dense degeneration was seen in all the photomicrographs that were analyzed. Counts revealed that the number of intact synapses per 100 μm^2^ remained comparable to un-operated *Wld*^*s*^ mice (Figure 3).

Although no terminals exhibited typical dark-dense degeneration, a few synapses had unusual morphologies that might represent early stages of degeneration. Some had washed out areas in the presynaptic terminal (Figure 4A, purple arrow) or empty vacuoles (Figure 2C, purple arrow); a few terminals had washed out areas with complex whorls of membranes, and sometimes membrane-bound sacs containing vesicles (Figure 4C). There were also a few profiles that appeared to be exceptionally large presynaptic terminals filled with synaptic vesicles (Figure 4B). Although uncommon (approximately one per 2,000 μm^2^), the appearance of these was quite striking.

The most obvious qualitative difference in the appearance of the neuropil in *Wld*^*s*^ mice at 4 days postlesion was that there were many spines exhibiting a very complex morphology with large chaliciform heads. Many spines had segmented PSDs (Figure 2C), often with finger-like protrusions (termed spinules) extending into the presynaptic terminal between the multiple PSDs (marked by red asterisks in Figure 2C). Unusually large, complex spine apparatuses were also evident (Figures 2C, 4D, SA). Figure 4D is a lower magnification view with spine heads colored yellow to illustrate the numerous spines with chaliciform heads in *Wld*^*s*^ mice. Spines with complex chaliciform heads, multiply segmented PSDs, spinules and large complex spine apparatuses were evident in the denervated zone in all *Wld*^*s*^ mice examined at 4 days postlesion. Indeed, these were such a distinguishing feature of the denervated neuropil of *Wld*^*s*^ mice that it was impossible to be blind as to the identification of micrographs from mutant mice. Spines with abnormal morphologies were even more evident at 8 days post-lesion (below).

A previous study reported spines with similar complex morphologies in the striatum 8–10 days after cortical lesions in *Wld*^*s*^ mice (Gillingwater et al., 2006). Of note, such complex spines were contacted by both degenerating and non-degenerating synapses. A caveat, however, is that synapses scored as “non-degenerating” that contact complex spines may be orphaned axons that not yet have begun to exhibit degenerative changes.

To further explore whether complex spine morphology is unique to synapses from orphaned axons that will eventually degenerate, we took advantage of the precise lamination of inputs to dentate granule cells. With complete lesions of the entorhinal cortex, approximately 95% of the synapses in the outer 2/3 of the molecular layer degenerate. However, proximal dendrites of dentate granule cells are innervated by commissural/associational axons, whose synapses do not degenerate. Assessment of spines in this zone revealed that some complex spines with chalice-shaped heads, spinules, segmented PSDs and large spine apparatuses were in fact present. To estimate the relative numbers of spines with unusual morphologies in the non-denervated inner molecular layer (IML) vs. the denervated outer molecular layer (OML), we collected sample micrographs at 3,000× from each zone (area of each image = 50 μm^2^) and counted spines with spinules or segmented PSDs. These counts revealed that although complex spines were present in the inner molecular layer, they were less common than in the denervated zone (3.27/100 μm^2^ in the IML vs. 9.56/100 μm^2^ in the OML).

Although spines in *Wld*^*s*^ mice exhibited changes indicating growth, dendrites appeared generally comparable to un-operated mice, and did not have the varicose appearance of dendrites contacted by degenerating terminals in WT C57Bl/6 mice.

### 8 Days Post-lesion

**FIGURE 5:**
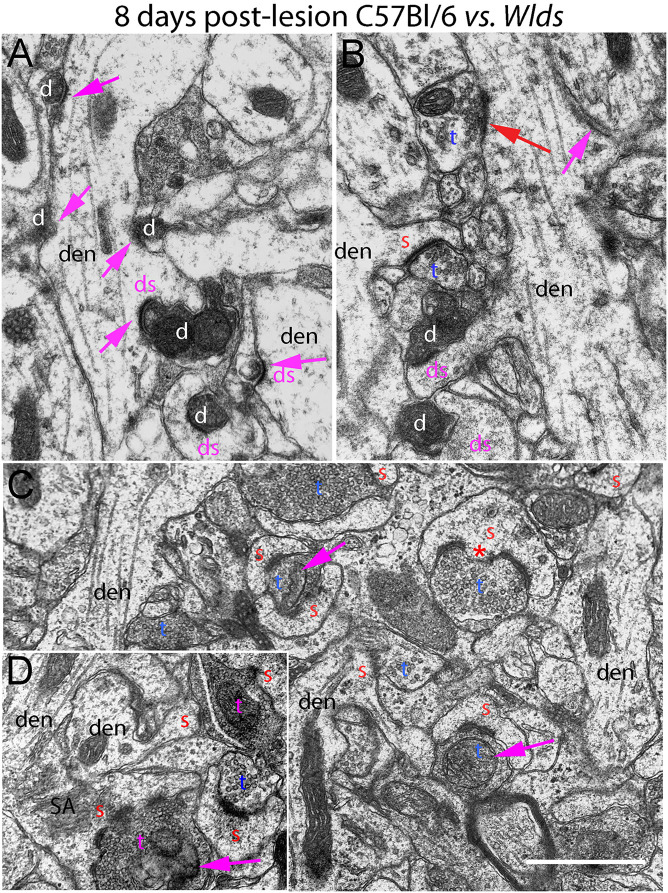
Ultrastructure of synapses at 8 days post-lesion in C57Bl/6 and *Wld*^*s*^ mice. **(A,B)** Dark-dense degeneration and collapsed spines at 8 days post-lesion in C57Bl/6 mice. Purple arrows indicate collapsed spines. Red arrow in **(B)** indicates non-degenerating synapse on a mound resembling a collapsed spine. **(C)** Non-degenerating synapses in the middle molecular layer of *Wld*^*s*^ mice at 8 days postlesion. **(D)** Very large spine with complex spine apparatus (SA) and multiply segmented PSD. Spinules are marked by red asterisks. SA, spine apparatus; red s, spine head contacted by non-degenerating synapse; blue t, non-degenerating synaptic terminal; white d degenerating synaptic terminal; purple ds, spine contacted by degenerating presynaptic terminal; den, dendrite; pr, polyribosomes. Calibration bar in **(A)** = 0.25 μm and applies to **(A–D)**.

At 8 days post-lesion the overall appearance of degenerating synapses in C57Bl/6 control mice was similar to what was seen at 4 days post lesion except that all degenerating terminals were dark and dense and none exhibited the electron lucent form of degeneration seen occasionally at 4 days (Figures 5A,B). Counts revealed that the number of degenerating synapses was comparable to 4 days whereas the number of intact synapses was slightly higher (Figure 3), indicating the early stage of reinnervation.

Of note, degenerating presynaptic terminals frequently contacted multiple spine heads. Degenerating terminals in contact with multiple spine heads has previously been described in rats (Steward and Vinsant, 1983). Our interpretation is that this configuration arises because presynaptic axons contract as they degenerate, pulling attached spines closer together.

As described previously in rats, some dark dense degenerating presynaptic terminals with attached spine heads were surrounded by astrocytes, identified as such by the characteristic appearance of the cytoplasm (electron lucent with few organelles, occasional glycogen granules, and intermediate filaments). It is likely that spine heads that appear to be engulfed by an astrocyte are still connected with the main dendrite via a spine neck that extends out of the plane of section.

As at 4 days, spines contacted by degenerating presynaptic terminals in C57Bl/6 mice had abnormal morphologies (cup-shaped heads and short amorphous spine necks) or had the appearance of fully collapsed spines (Figures 5A,B). Empty PSDs were more common at 8 days post-lesion than at 4 days, but were still relatively uncommon. Dendritic shafts had a varicose appearance as at 4 days.

Some normal-appearing presynaptic terminals were scattered amongst the degenerating synapses, and some of these contacted mounds on the dendrite, inviting the speculation that they were new connections onto collapsed spines that had been cleared of degeneration debris (red arrow, Figure 5B).

**FIGURE 6:**
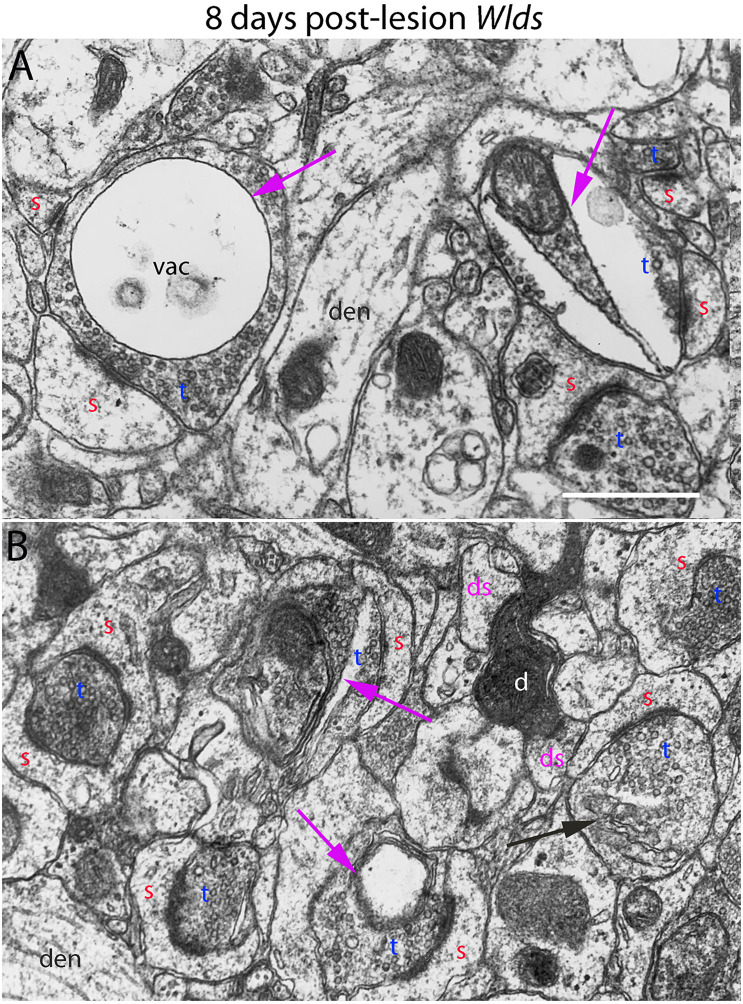
Ultrastructural abnormalities of synapses at 8 days post-lesion in *Wld*^*s*^ mice. **(A)** Terminals exhibiting abnormalities as vacuoles and membrane-delimited vacuoles surrounded by areas of essentially normal-looking presynaptic cytoplasm with vesicles. **(B)** A rare example of a terminal exhibiting dark-dense degeneration amongst other synapses on spine heads with chaliciform morphologies. Red s (red), spine head; t (blue), non-degenerating synaptic terminal; d (white) degenerating synaptic terminal; ds (purple) spine contacted by degenerating presynaptic terminal; den, dendrite; pr, polyribosomes. Calibration bar in **(A)** = 0.25 μm and applies to **(A,B)**.

At 8 days post-lesion in *Wld*^*s*^ mice, most presynaptic terminals still appeared relatively normal (Figures 5C, 6), but some terminals did exhibit abnormal morphologies. Some terminals that looked relatively normal otherwise contained unusual looking membrane structures that were not readily identifiable as typical organelles (Figure 5C, purple arrows). Some terminals exhibited abnormalities with some similarity to the lucent form of degeneration in WT mice. In contrast to typical lucent degeneration, however, where the entire terminal appears swollen and washed out, some terminals *Wld*^*s*^ mice had large membrane-delimited vacuoles surrounded by areas of essentially normal-looking presynaptic cytoplasm with vesicles (Figure 6A, vac) or washed out regions along with areas containing cytoplasm and vesicles (right side, Figure 6A). These profiles were unlike anything seen in WT C57BL/6 mice and as such, were another distinguishing feature of *Wld*^*s*^ mice. Still other terminals had a dark cytoplasm with embedded vesicles that could represent an early stage in the transition to dark-dense degeneration. Very few presynaptic terminals exhibited typical dark-dense degeneration, however (Figure 6B).

In contrast to the denervated spines in C57Bl/6 mice, in *Wld*^*s*^ mice, there were very few profiles resembling collapsed spines. Instead, as at 4 days post-lesion, spines with hypertrophied caliciform heads were prominent. Some spines exhibited even more dramatic hypertrophy than at 4 days post-lesion, exhibiting complex heads bearing multiple PSDs, spinules extending into the presynaptic terminal and an elaborate spine apparatus at the base. Figure 5D illustrates a spine with 6 separate PSD segments and a large spine apparatus (SA).

### 12 Days Post-lesion

**FIGURE 7:**
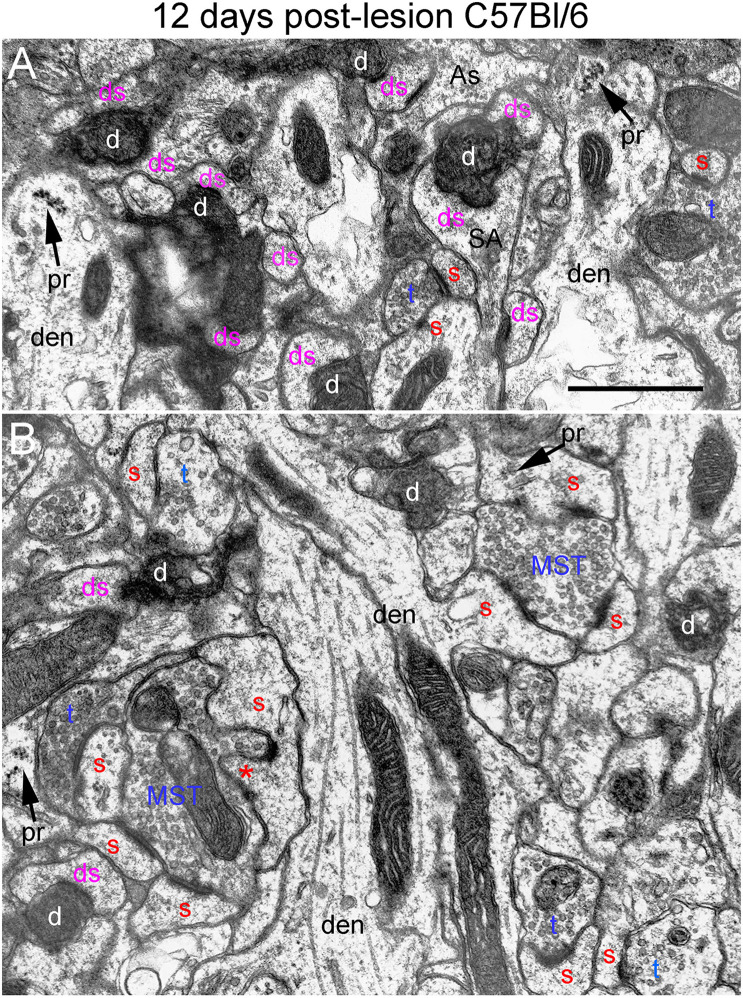
Ultrastructure of synapses at 12 days post-lesion in C57Bl/6 mice. **(A)** Dark-dense terminal contacting multiple spine heads in C57Bl/6 mice. Arrows indicate empty PSDs (not contacted by either an intact or degenerating presynaptic terminal). Upper arrow indicates empty PSD apposed by astrocyte process. **(B)** Non-degenerating presynaptic terminal contacted multiple PSDs. Red s (red), spine head; t (blue), non-degenerating synaptic terminal; d (white) degenerating synaptic terminal; ds (purple) spine contacted by degenerating presynaptic terminal; den, dendrite; pr, polyribosomes. Calibration bar in **(A)** = 0.25 μm and applies to **(A,B)**. *spinule.

At 12 days postlesion, the denervated zone of control C57Bl/6 mice still contained large numbers of synaptic terminals exhibiting dark-dense degeneration. Counts revealed that the number of degenerating synapses was comparable to 4 and 8 days. As at 8 days, many of the dark-dense terminals contacted multiple spine heads forming nests of denervated spine heads with a degenerating terminal at the center (Figure 7A). Empty PSDs (not contacted by either an intact or degenerating presynaptic terminal) were also evident (Figure 7A); some of these were apposed by astrocyte processes.

Qualitatively, it was clear that there were a larger number of non-degenerating synapses than at 8 days. Indeed, the number of intact synapses was about twofold higher than at 8 days (Figure 3) indicating formation of new synapses between 8 and 12 days. A distinguishing feature of the neuropil at 12 days post-injury was that many of the non-degenerating presynaptic terminals contacted multiple PSDs (Figure 7B). Multiple synapse terminals (MSTs) are a common feature of reinnervating axons in this model (Steward et al., 1988b), and a possible explanation for this configuration is that sprouting axons grow into the nests of denervated spine heads where they form multiple synaptic connections.

To determine the extent of increases in multiple synapse terminals, we counted presynaptic terminals contacting 2 or more PSDs and expressed the counts as numbers of MSTs per 1,000 μm^2^ of neuropil. Confirming our qualitative impression, the number of MSTs was in fact almost threefold higher at 12 days post lesion than at 4 days (see below for additional quantitative analyses).

Spines contacted by degenerating presynaptic terminals continued to exhibit the atrophic morphologies described above. Spine necks were irregular in shape and many had the morphology of collapsed spines, indicating spine atrophy, and in some cases collapse or withdrawal of the spine into the parent dendrite. However, some non-degenerating synapses were on spines with a morphology similar to un-injured mice (Figure 7B) suggesting restoration of spine morphology with reinnervation. Polyribosome clusters were evident in dendrites and sometimes spine heads, consistent with previous studies documenting increases in spine-associated polyribosomes during reinnervation (Steward, 1983).

**FIGURE 8:**
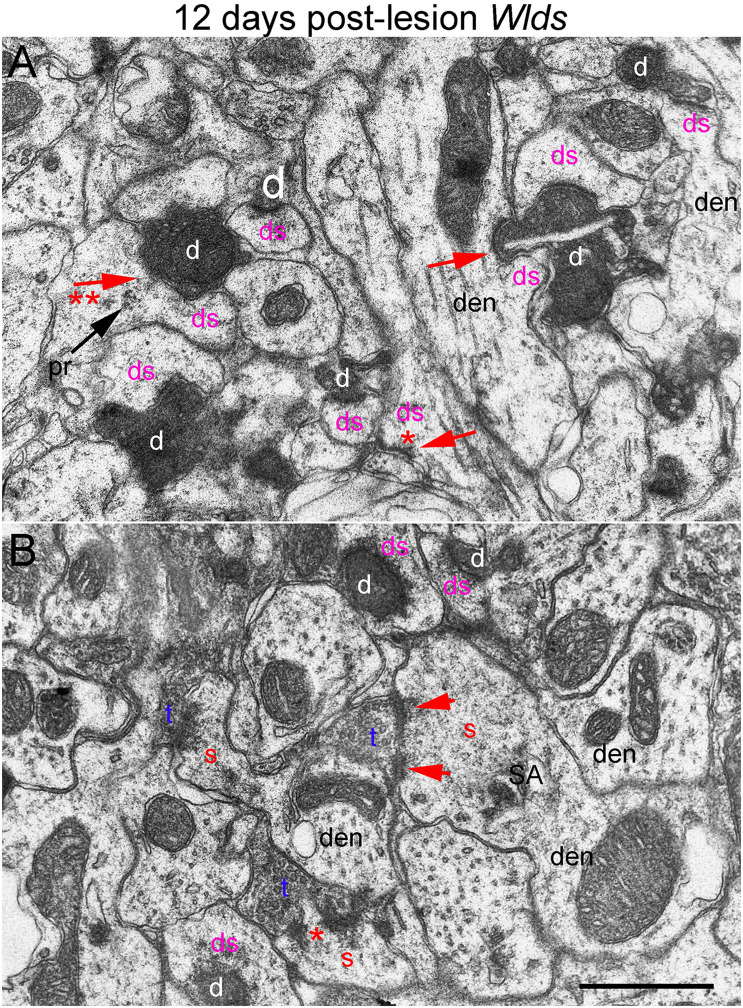
Ultrastructural abnormalities of synapses at 12 days post-lesion in *Wld*^*s*^ mice. **(A)** Synaptic terminals exhibiting dark-dense degeneration and collapsed spines at 12 days post-lesion in *Wld*^*s*^ mice. Note slit in the large dark-dense terminal on the center right. Arrow with 2 asterisks indicates spine with segmented PSD and polyribosomes (pr) contacted by a degenerating presynaptic terminal. **(B)** Large complex spines with segmented PSDs (short arrows) and spinule (asterisk). Red s (red), spine head; t (blue), non-degenerating synaptic terminal; d (white) degenerating synaptic terminal; ds (purple) spine contacted by degenerating presynaptic terminal; den, dendrite; pr, polyribosomes. Calibration bar in **(A)** = 0.25 μm and applies to **(A,B)**.

In *Wld*^*s*^ mice at 12 days postlesion, degenerating synaptic terminals were more numerous than at 8 days, although there were still not as many as in C57Bl/6 mice (Figure 3). Degenerating terminals exhibited dark-dense degeneration (Figure 8), but the terminals weren’t as dark and dense as in WT mice and synaptic vesicles and mitochondria could still be discerned in the dark dense presynaptic cytoplasm. Some terminals exhibiting dark-dense degeneration had the long slits that were frequently seen in terminals of Wld^*s*^ mice at 8 days. Counts revealed decreases in the number of intact synapses in comparison to 8 days post-lesion to a level that was comparable to WT C57Bl/6 mice (Figure 3).

Most spines contacted by degenerating presynaptic terminals were short and stubby and there were some profiles suggestive of collapse or withdrawal of the spine into the dendrite (Figure 8A, arrows; asterisk indicates a collapsed spine that is not contacted by either an intact or degenerating presynaptic terminal). Thus, although the initial response at 4–8 days post-lesion in *Wld*^*s*^ mice is paradoxical hypertrophy of spines contacted by terminals of orphaned axons, spine atrophy does eventually occur in parallel with the onset of degeneration of orphaned presynaptic terminals.

Large complex spines with segmented PSDs were still present at 12 days post-lesion (Figure 8B), but these were less complex and less numerous than at 8 days and also had a more amorphous appearance than at 4 and 8 days suggesting that they were beginning to undergo atrophy and collapse. Also, most of the large complex spines that were present were contacted by presynaptic terminals that were not exhibiting degenerative changes.

### 16 Days Post-lesion

At 16 days postlesion, the denervated zone of control C57Bl/6 mice appeared qualitatively similar to what was seen at 12 days post-lesion with large numbers of synaptic terminals exhibiting dark-dense degeneration (not shown). Counts revealed that the numbers of degenerating and intact synapses were comparable to those at 12 days (Figure 3), suggesting minimal removal of degenerating synapses or additional formation of new synapses.

**FIGURE 9:**
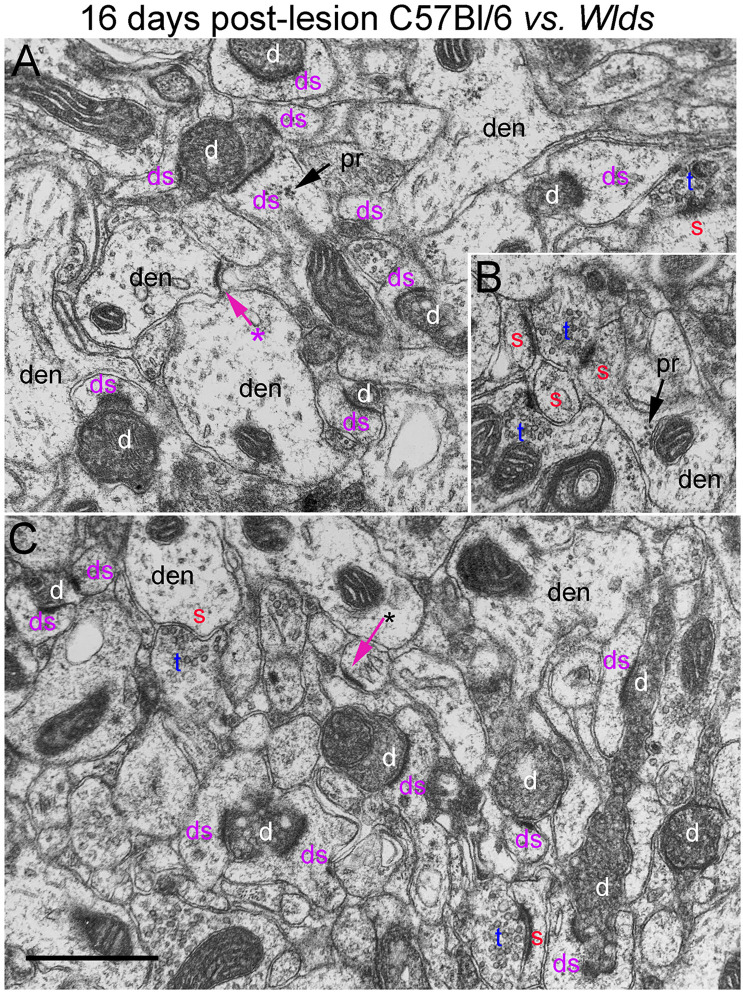
Ultrastructure of synapses at 16 days post-lesion in C57Bl/6 and *Wld*^*s*^ mice. **(A)** Dark-dense terminal contacting multiple spine heads in C57Bl/6 mice. Arrows indicate empty PSDs (not contacted by either an intact or degenerating presynaptic terminal). Upper arrow indicates empty PSD apposed by astrocyte process. **(B)** Non-degenerating presynaptic terminal contacted multiple PSDs. **(C)** Terminals exhibiting dark-dense degeneration at 16 days post-lesion in *Wld*^*s*^ mice. Red s (red), spine head; t (blue), non-degenerating synaptic terminal; d (white) degenerating synaptic terminal; ds (purple) spine contacted by degenerating presynaptic terminal; den, dendrite; pr, polyribosomes. Calibration bar in **(C)** = 0.25 μm and applies to **(A–C)**. *empty PSD.

In *Wld*^*s*^ mice at 16 days postlesion, synaptic terminals in the denervated portion of the molecular layer exhibited dark-dense degeneration in numbers that were comparable to C57Bl/6 mice (Figure 9 and for counts, see Figure 3). Empty PSDs were also present (Figure 9 indicated by asterisk). The number of non-degenerating synaptic terminals was comparable to C57Bl/6. Some of these were MSTs (Figure 9B). As was the case at 4 days post-lesion in C57Bl/6 mice, some degenerating presynaptic terminals contacted multiple spine heads (Figure 9A, upper right, Figure 9B, lower middle).

Non-degenerating synaptic terminals that appeared entirely normal were intermixed with dark-dense synaptic terminals; some of these intact terminals were MSTs (Figure 9B). Counts revealed that the number of non-degenerating terminals was comparable to C57Bl/6 and slightly higher than the number at 12 days post-lesion in *Wld*^*s*^ mice (Figure 3).

### Quantification of Synapse Parameters

**FIGURE 10:**
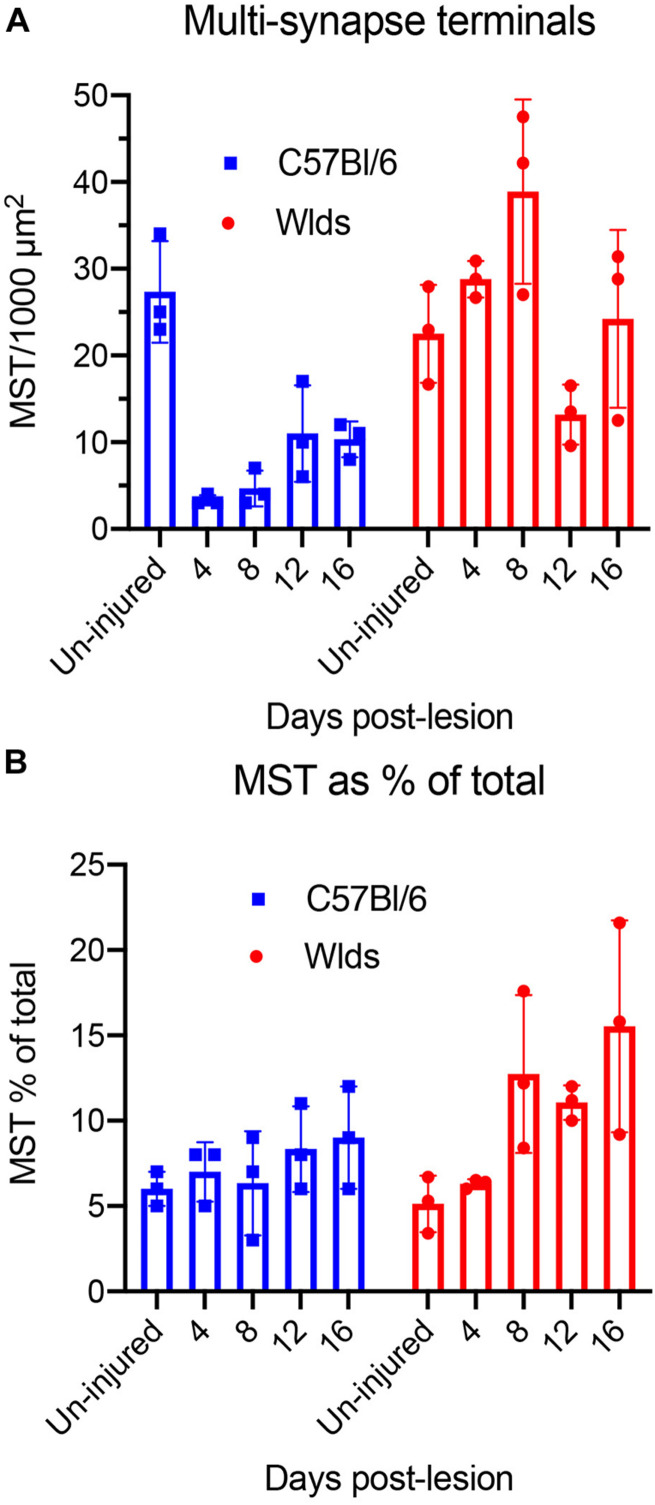
Quantification of synapse parameters. **(A)** Counts of multiple synapse terminals (MSTs) contacting 2 or more PSDs per 1,000 μm^2^ of neuropil. 2-way ANOVA revealed significant differences between genotypes [*F*(1,4) = 39.2, *p* = 0.003], and significant differences over days post-lesion [*F* = 4.57, *p* = 0.047]. **(B)** Percent of presynaptic terminals that were MSTs. 2-way ANOVA revealed significant differences between genotypes [*F*(1,4) = 9.57, *p* = 0.036], whereas differences over days post-lesion were not significant [*F* = 4.27, *p* = 0.072].

We carried out additional analyses to quantify changes in synapse parameters (Figure 10). First, to quantify multiple synapse terminals (MSTs), we counted presynaptic terminals contacting 2 or more PSDs and expressed the values as counts per 1,000 μm^2^ of neuropil (Figure 10). In C57Bl/6 mice, the number of MSTs decreased at 4 days post-lesion to about 5% of the values in un-injured mice in parallel with the decreases in the number of intact synapses, then increased gradually over the post-lesion interval in parallel with re-appearance of non-degenerating synapses. In *Wld^s^* mice, however, the number of MSTs was higher at 4 days post-lesion than in un-injured mice, increased further at 8 days. Numbers of MSTs decreased at 12 days to about 50% of un-operated control in parallel with the appearance of degenerating presynaptic terminals, and then increased again at 16 days post-lesion to an average comparable to un-operated mice (although with considerable variability between cases). Statistical analysis by 2-way ANOVA revealed significant differences between genotypes [*F*(1,4) = 39.2, *p* = 0.003], and significant differences over days post-lesion [*F* = 4.57, *p* = 0.047].

We also calculated the percent of presynaptic terminals that were MSTs (Figure 10B). In uninjured mice of both strains, about 6% of the presynaptic terminals were MSTs. In C57Bl/6 mice, MSTs increased slightly to about 8% of control values by 16 days. In *Wld*^*s*^ mice, the percent of presynaptic terminals that were MSTs increased to approximately threefold higher than control by 16 days. Statistical analysis by 2-way ANOVA revealed significant differences between genotypes [*F*(1,4) = 9.57, *p* = 0.036], whereas differences over days post-lesion were not significant [*F* = 4.27, *p* = 0.072].

## Discussion

The goal of this study was to further explore the curious phenotypes of the *Wld*^*s*^ mutation. Although the mutation was discovered over 3 decades ago and the mutant protein has been identified as a fusion protein made up of the amino terminal 70 amino acids (N70) fragment of ubiquitination factor U4B, the complete coding region of NAD+ synthesizing enzyme nicotinamide adenyltransferase 1 (NMNAT1), and an 18 amino acid linking region, the cell biology that underlies the signature phenotype remains mysterious. Most previous studies have focused on Wallerian degeneration of axons, particularly in the peripheral nervous system, but to our knowledge, there have been only two previous studies focusing on consequences of the mutation on degeneration of CNS synapses (Gillingwater et al., 2006; Wright et al., 2010).

Here, we were particularly interested in the timing and nature of degeneration of presynaptic terminals of orphaned axons and on the postsynaptic side, how atrophy of postsynaptic spines is affected. Electron microscopy can provide insights into cell biological mechanisms, and provide hints about whether the mutation mainly affects axon degeneration or also confers a degree of protection on the post-synaptic side, preventing or delaying spine atrophy or causing other un-predicted alterations in spines. Accordingly, our quantitative electron microscopic analysis focused on: (1) The timing of degeneration of orphaned synaptic terminals in *Wld*^*s*^ mice; (2) changes in spines contacted by orphaned synapses, and especially whether in this model of lesion-induced synapse plasticity, spines in *Wld*^*s*^ mice exhibited the remarkable changes in ultrastructure previously reported in the striatum following cortical lesion (Gillingwater et al., 2006); (3) ultrastructural signs of reinnervation.

### Timing of Degeneration of Orphaned Synaptic Terminals

A previous study of consequences of the mutation on degeneration of CNS synapses (Gillingwater et al., 2006) documented delayed onset of degenerative changes in cortico-striate synapses after cortical injury. However, this study did not assess time points beyond 10 days post-injury. Also, a technical caveat of this previous study was that in the quantitative analysis of synapse numbers over time, counts were expressed as numbers of synapses observed during a 15 min observation period on the electron microscope. Expressing data in this way does not allow comparisons across labs because the counts depend on how much area was assessed during the 15 min scans.

Our results demonstrate that onset of degeneration of presynaptic terminals of the perforant path is similarly delayed by many days in *Wld*^*s*^ mice. There are minimal signs of degenerative changes in presynaptic terminals at 4 days post-injury at a time when amputated axons in WT rats and mice exhibit full-blown degeneration. Even at 8 days post-lesion, most presynaptic terminals of orphaned axons in *Wld*^*s*^ mice still appear relatively normal, although some exhibit ultrastructural abnormalities that might represent previously uncharacterized early degenerative changes including large, elongated membrane bound areas with empty lumens and atypical membrane structures. Typical presynaptic degeneration does eventually occur, however. At 12 days, some terminals exhibit dark-dense degeneration, although many terminals still appear relatively normal but at 16 days post-injury, synaptic terminals of orphaned axons exhibit the full extent of degeneration seen in WT C57Bl/6 mice.

The fact that typical signs of synaptic degeneration do eventually occur in *Wld*^*s*^ mice suggests that the mutation does not alter the actual mechanisms of synapse degeneration, and instead maintains the synapse in a viable state. It is unknown how such synaptic terminals of orphaned axons operate physiologically. The presence of large numbers of synaptic vesicles suggests that orphaned terminals may remain capable of packaging neurotransmitter, but it is unknown whether the terminals actually release neurotransmitter onto the postsynaptic neuron when there is no cell body to generate action potentials. Indeed, the physiology of orphaned axons and their terminals is essentially un-explored, except that peripheral nerve axons do remain capable of propagating action potentials when stimulated electrically (Perry et al., 1991).

### Mechanisms Underlying Spine Hypertrophy

Presynaptic degeneration of amputated axons in WT C57Bl/6 mice is accompanied by withering and collapse of spines as previously described in rats (Steward and Vinsant, 1983). In striking contrast, spines contacted by synapses of orphaned axons exhibited dramatic hypertrophy and remodeling of post-synaptic membrane specializations (PSDs). Hypertrophy was evident at 4 days post-lesion before the onset degenerative changes in orphaned presynaptic terminals and was most dramatic at 8 days. By 12 days, spines began to exhibit collapse and withering. The dramatic hypertrophy and PSD remodeling indicates a robust and paradoxical spine growth in *Wld*^*s*^ mice during the time period when denervated spines in WT mice are withering and retracting. Similar dramatic spine hypertrophy was reported by Gillingwater et al. (2006) in their study of cortico-striate synapse degeneration, and our findings extend the story by showing that the spine hypertrophy is transient.

### *Trans*-Synaptic Signaling From Orphaned Axons

The remarkable hypertrophy of spines on neurons contacted by terminals of orphaned axons suggests that orphaned presynaptic terminals must somehow signal to the postsynaptic neuron. The nature of the signaling is unknown, however. The alterations in spine morphology including transformation from simple to caliciform morphology, PSD segmentation and appearance of spinules are remarkably similar to alterations that occur as a result of induction of long-term potentiation of perforant path synapses (Desmond and Levy, 1983, 1988). Very similar changes have been reported as a result of other interventions that induce intense synaptic activity (Petralia et al., 2021). These alterations are paradoxical in the present setting because the spines exhibiting this transformation are contacted by orphaned axons that have been separated from their cell bodies. Orphaned axons appear morphologically intact and do contain large numbers of synaptic vesicles, so it is conceivable that they continue to release neurotransmitter. However, action potentials are normally generated at the cell body or initial segment, which was removed by the aspiration lesion. If spine changes are induced by intense presynaptic activity, then the activity must somehow be generated intrinsically in the orphaned axons. To our knowledge, there have been no studies of spontaneous action potential generation by orphaned axons before they begin to degenerate. If this is the mechanism, it would represent another remarkable consequence of the *Wld*^*s*^ mutation on axonal pathophysiology.

One possibility is that silencing of action potential-driven neurotransmitter release without degeneration is a signal for spine hypertrophy. This idea is counter to the prevailing concept that spines are maintained by synaptic activity and decreases in activity typically lead to decreases rather than increases in spine size. Another possibility is that surviving orphaned axons release neurotransmitter with different dynamics than normal, and that this abnormal release is the critical signal. A third possibility is that preserved terminals of orphaned axons in *Wld*^*s*^ mice release some other molecule that triggers spine growth. These questions indicate that there are still many unknowns about the cell biological consequences of the *Wld*^*s*^ mutation.

The postsynaptic mechanisms underlying the dramatic hypertrophy of spines are unknown. Spine hypertrophy could be a manifestation of the *Wld*^*s*^ mutation on the intrinsic cell biology of the postsynaptic cell, altering its responses to impending degeneration of presynaptic axons. Alternatively, if the effect of the mutation is limited to delaying Wallerian degeneration of axons and their synaptic terminals, then postsynaptic hypertrophy could be due to some signal(s) from the orphaned axons during the time that they are protected from degeneration. Our results do not provide a definitive answer but do provide a basis for formulation of questions.

One question is whether hypertrophy is limited to spines contacted by preserved orphaned axons or whether it is a general postsynaptic response involving all spines including those contacted by un-injured axons. A previous study that assessed synaptic degeneration in the striatum up to 10 days after cortical lesions reported that very complex spines on striatal neurons contacted by both degenerating and non-degenerating synapses (Gillingwater et al., 2006). A caveat, however, is that synapses scored as non-degenerating that contact complex spines may be orphaned axons that not yet have begun to exhibit degenerative changes. In this case, the complex morphology seen in the study of cortico-striatal synapses may still be unique to synapses from orphaned axons that will eventually degenerate.

Definitive identification of spines contacted by synaptic terminals of orphaned axons is less of a complication in the present study because about 95% of the synapses in the outer molecular layer of the dentate gyrus derive from the ipsilateral entorhinal cortex, and thus will eventually degenerate following complete entorhinal cortex lesions. The inner molecular layer of the dentate gyrus contains synapses of the dentate commissural/associational system, which do not degenerate, and there is essentially no overlap in the terminal fields of these two afferent systems. Nevertheless, spines with complex morphology were evident in the non-denervated inner molecular layer of *Wld*^*s*^ mice supporting the conclusion that there is hypertrophy of non-denervated spines.

Although a limitation of the present study is the use of a single technical approach (electron microscopy), our findings do point to questions for future studies. For example, the dramatic hypertrophy of spines suggests some sort of *trans*-synaptic signaling that could be related to neurotransmitter release by orphaned axons or release of some other signaling molecule. It would be informative to create chimeras where presynaptic neurons were from one strain and postsynaptic neurons were from another. The question would be whether postsynaptic neurons from *Wld*^*s*^ mice exhibited spine hypertrophy when there was degeneration of synaptic contacts from WT mice. If future studies indicate that hypertrophy is not an intrinsic effect of the mutation on the postsynaptic neuron, then identification the *trans*-synaptic signal is of great interest and could suggest new ways to protect postsynaptic neurons from denervation-induced atrophy. It would also be of great interest to assess whether other unique ultrastructural features, especially spine hypertrophy, were seen in other strains of mice with delayed Wallerian degeneration (SARM1 knockout mice for example).

### Ultrastructural Signs of Reinnervation in *Wld*^*s*^ vs. C57Bl/6 Mice

In the model system of reinnervation of dentate granule cells following entorhinal cortex lesions, synapse replacement is reflected by loss and then replacement of non-degenerating synapses over time. There is no categorical marker or definitive morphology to identify new synapses, although tracing studies have demonstrated that some are multi-synapse terminals (Steward et al., 1988b). Defining synapse replacement in *Wld*^*s*^ mice is not straightforward because synapse loss is delayed, extending into the early phase of new synapse formation in WT C57Bl/6 mice between 4 and 8 days post-lesion. At 8 days post-lesion in *Wld*^*s*^ mice, most synapses that will eventually degenerate have not yet begun to exhibit degenerative changes. This mix of orphaned synapses that will eventually degenerate and new synapses compromises EM quantification of the time course of new synapse formation. By 12 days, the numbers of intact synapses and degenerating synapses in *Wld*^*s*^ mice are equivalent to what is seen in C57Bl/6 mice, and then numbers of intact synapses increase between 12 and 16 days. These data are consistent with previous studies of sprouting of different inputs to dentate granule cells after entorhinal cortex lesions. Sprouting of septal cholinergic axons is delayed and less extensive in *Wld*^*s*^ mice (Steward et al., 1988b; Shi and Stanfield, 1996). Sprouting of commissural axons is delayed to an even greater extent than sprouting of cholinergic axons with the first sign of sprouting being at 12 days post-lesion (Shi and Stanfield, 1996).

### Implications for Searches for Naturally-Occurring Neuroprotective Mutations

The *Wld*^*s*^ mutation was discovered during studies of axonal pathophysiology, specifically Wallerian degeneration of peripheral nerve axons. It is highly unlikely that this spontaneous mutation would have been noticed otherwise because there are to our knowledge no reports of any phenotype in un-injured mice with the *Wld*^*s*^ mutation. Discovery of the *Wld*^*s*^ mutation happened in much the same way as our discovery of other murine genetic backgrounds that confer protection against excitotoxic neurodegeneration (Schauwecker and Steward, 1997). The initial surprising discovery was an unexpected finding from studies of hippocampal degeneration following kainic acid-induced seizures, which revealed that a commonly used inbred strain (C57Bl/6) was highly resistant to kainic acid-induced neurodegeneration (Schauwecker and Steward, 1997). One wonders how many other naturally-occurring mutations have neuroprotective phenotypes that are only manifested in the context of pathophysiology.

## Data Availability Statement

The raw data supporting the conclusions of this article will be made available by the authors, without undue reservation, to any qualified researcher.

## Ethics Statement

The animal study was reviewed and approved by Institutional Animal Care and Use Committee at the University of Virginia.

## Author Contributions

OS planned the study, analyzed the data, and wrote the manuscript. PF and JY prepared EM samples and contributed to writing the manuscript. All authors contributed to the article and approved the submitted version.

## Conflict of Interest

OS is a co-founder and has economic interests in the company “Axonis Inc.”, which holds a license on patents relating to axon regeneration. The remaining authors declare that the research was conducted in the absence of any commercial or financial relationships that could be construed as a potential conflict of interest.

## Publisher’s Note

All claims expressed in this article are solely those of the authors and do not necessarily represent those of their affiliated organizations, or those of the publisher, the editors and the reviewers. Any product that may be evaluated in this article, or claim that may be made by its manufacturer, is not guaranteed or endorsed by the publisher.
